# Extreme weather events and mental health impacts in West Africa: A systematic review of evidence gaps

**DOI:** 10.4102/jphia.v17i1.1760

**Published:** 2026-09-10

**Authors:** Jamaji C. Nwanaji-Enwerem, Joshua M. Perez-Cruet, Nadav Sprague, Stephen Uong, Caroline Obi, Shenita Peterson, Adetoun Mustapha, Max Jordan Nguemeni Tiako, Saria Hassan, Yun Hang, Temilola Oluseyi, Lisa Reyes Mason, Christine Ekenga

**Affiliations:** 1 Department of Emergency Medicine, School of Medicine, University of Pennsylvania, Philadelphia, Pennsylvania, United States of America; 2 Department of Medicine, School of Medicine Washington University, St. Louis, Missouri, United States of America; 3 Department of Environmental Health, School of Public Health, Columbia University, New York, New York, United States of America; 4 Department of Public Health, Georgia Department of Public Health, Atlanta, Georgia, United States of America; 5 Department of Environmental Health, School of Public Health, Emory University, Atlanta, Georgia, United States of America; 6 Department of Medicine, Faculty of Medicine, Nigerian Institute of Medical Research, Lagos, Nigeria; 7 Department of Medicine, University of California, Los Angeles, California, United States of America; 8 Department of Environmental Health, Faculty of Public Health, University of Texas Health Science Center at Houston, Houston, Texas, United States of America; 9 School of Social Work, University of Denver, Denver, Colorado, United States of America

**Keywords:** climate health, stress, well-being, flooding, droughts

## Abstract

**Background:**

A growing body of evidence, primarily from high-income nations, suggests that extreme weather events can have a profound impact on mental health.

**Aim:**

To contribute to a more inclusive understanding of this public health threat, we reviewed the literature to understand the impact of climate-related extreme weather events on mental health in the West African context.

**Setting:**

This study is a systematic review of studies based in West Africa.

**Methods:**

Our literature search of six databases (PubMed/MEDLINE, Index to Nursing and Allied Health Literature [CINAHL], EMBASE, PsycINFO, Cochrane and Web of Science) from January 2007 to December 2024 identified 2968 articles of which 13 met study inclusion criteria.

**Results:**

These 13 studies were predominantly cross-sectional and qualitative. The studies took place in four West African countries (Burkina Faso, Ghana, Nigeria and Sierra Leone) and reported direct and indirect relationships between climate-related weather events and poor mental health. The articles also highlighted that certain groups like women and persons of low socioeconomic status are likely to experience more adverse mental health impacts due to gender roles and factors associated with a lower social standing. The studies further suggest bolstering population climate health education, reforming building and development mechanisms, and targeting social programmes to women and other vulnerable groups as potential strategies to address the negative impact of climate on mental well-being.

**Conclusion:**

Findings from this study as well as continued investments in mental health clinical capacity will be important for meeting existing shortfalls in mitigating the impact of climate change on mental health.

**Contribution:**

There is a need for greater research investment in larger studies, in more West African countries, and including substantial proportions of the population like children.

## Introduction

Extreme weather events, such as floods, typhoons, heatwaves and droughts, are becoming more frequent and severe due to climate change, with profound and often lasting effects on human health and well-being.^1,2^ A large and growing body of evidence, particularly from high-income countries, suggests that the mental health impacts of extreme weather are profound and multifaceted.^3,4,5^ The Intergovernmental Panel on Climate Change’s (IPCC) Fourth Assessment Report, noteworthy for its expanded focus on the health impacts of climate change, recognised that exposure to extreme weather events such as hurricanes, floods or heatwaves can lead to psychological stress and trauma, affecting mental well-being.^6^ An extreme weather event can contribute to heightened levels of anxiety and depression, stemming from the uncertainty and fear associated with both the immediate threat and the long-term challenges associated with the event.^1,6,7^ The loss of livelihoods, homes, possessions and sometimes family members or friends can trigger intense grief, and the grieving process may be prolonged and complicated by the unique circumstances of the event. Moreover, displacement, whether temporary or permanent, can induce significant stress as forced relocation, loss of community ties, and the challenges of adapting to a new environment all contribute to the hardships faced by those affected by extreme weather disasters.^3,5^

In this work, we focus on the West African region. West Africa, characterised by its diverse climate and topography, specifically faces a range of extreme weather events that have significant implications for the well-being of its population.^1,8,9,10^ In this region, droughts are persistent challenges, resulting in water scarcity, agricultural difficulties and food insecurity. Extended periods without rainfall can lead to crop failures, impacting the livelihoods of those heavily reliant on agriculture. The recurrent nature of droughts not only exacerbates economic vulnerabilities, but also poses a sustained threat to food security, contributing to economic hardships for communities that rely on agriculture and natural resources for their sustenance. In contrast to droughts, floods pose immediate and impactful threats as heavy rainfall often brings the risk of flash floods and river overflows. The consequences of flooding are far-reaching and include the displacement of communities and widespread damage to infrastructure and homes. The varied extreme weather events in West Africa, from droughts and floods to tropical cyclones, collectively contribute to a complex set of challenges that can impact the mental health of the region’s residents.

Climate-related challenges, including those impacting mental health, transcend national borders, making a regional lens useful. A regional approach allows us to account for shared environmental conditions and sociopolitical contexts across countries, while also broadening the population to which our review is directly applicable. Moreover, this regional focus is solutions-oriented, aligning with established mechanisms for cross-national collaboration, such as the Economic Community of West African States (ECOWAS), which has a proven track record of supporting regional health initiatives. Efforts like the ECOWAS Medicines Regulatory Harmonisation initiative offer valuable models for coordinated responses to emerging challenges,^11^ including those at the intersection of climate change and mental health.

Mental health disorders remain a significant and often underreported challenge in West Africa with studies estimating lifetime prevalences of mental illness ranging from 1% to 15.8%.^12^ Despite this substantial burden, access to mental healthcare remains critically limited. For instance, in 2009, it was estimated that 98% of Sierra Leoneans with mental illness were not receiving treatment, in part due to a lack of public psychiatric facilities outside of the capital.^13^ Reports from Liberia indicate that there are only a handful of psychiatrists for its population of over 4 million.^14^ These findings agree with data showing that over 90% of people living with mental illness are untreated in low- and middle-income countries, including West African nations, as compared to 44% – 70% in high-income countries.^15^ In addition to a lack of qualified mental health professionals, several other barriers contribute to this treatment gap including stigma, cultural perceptions, infrastructural deficiencies and economic constraints.^16,17,18^ For example, Gambia and Ghana allocate 0.5% and 1.3% of their health budget to mental health, respectively.^19^ These allocations are well below international recommendations and benchmarks for mental health investment in low- and middle-income countries.^20,21,22^ Given the limitations in existing mental healthcare, understanding the underlying contributors to mental health disorders is increasingly urgent. In this context, characterising the relationship between extreme weather events and mental health is especially important, as climate-related stressors may significantly contribute to the region’s mental health burden. As efforts continue to strengthen healthcare systems, identifying and addressing these climate-related factors could help narrow the mental health treatment gap by reducing the incidence and severity of mental health conditions.

We conducted a systematic review of the existing peer-reviewed literature to summarise and characterise the mental health impacts of extreme weather events in West Africa. By evaluating existing research, we aimed to identify knowledge gaps and inform future research priorities that will enable public health and clinical practitioners to make informed decisions and formulate policies aimed at mitigating the mental health impacts of climate change in the region.

## Methods

### Search strategy

The review followed the Preferred Reporting Items for Systematic Reviews and Meta-Analyses (PRISMA) guidelines for reporting systematic reviews.^23^ The senior author and a health sciences librarian searched six electronic databases to find scholarly articles published in peer-reviewed journals between 01 January 2007 (the release year of the IPCC’s Fourth Assessment Report) and 31 December 2024. These databases included PubMed/MEDLINE, Index to Nursing and Allied Health Literature (CINAHL), EMBASE, PsycINFO, Cochrane and Web of Science. Search terms were developed in consultation with the health sciences librarian. For this review, we were guided by the IPCC definition of extreme weather events as rare events at a particular place and time, including but not limited to floods, droughts and extreme temperatures. Search terms were developed to broadly align with this framework and capture a wide range of relevant climate-related exposures.^24,25^ Key extreme weather event search terms included weather, climate, global warming, disaster, extreme event, extreme heat, extreme temperature, heat wave, heatwave, wildfire, wild fire, drought, flood, heavy precipitation, heavy rain, hurricane, landslide, mudslide, tidal wave, tropical storm, tsunami, typhoon, cyclone, monsoon, thermal, tornado, greenhouse, environmental impact and natural disaster.

Mental health search terms were selected based on existing global health literature and aimed to capture a broad range of psychological outcomes, including general mental health constructs, specific psychiatric disorders, trauma-related conditions and behavioural symptoms. This inclusive approach was intentional given heterogeneity in mental health definitions, measurement tools and cultural expressions of distress across settings.^26^ Key mental health search terms included mental health, mental illness, mental disorders, wellbeing, well-being, psych, post-traumatic, post-traumatic stress disorder (PTSD), psychiatric, psychosis, anxiety, angst, anxious, depression, schizophrenia, bipolar, bi-polar, suicide, stress, resilient, mood, aggression, violent, self-injury, self-harm, abuse, substance, alcohol, violence and behavioural symptoms. See Online Appendix Table 1-OA1 for the full search strategy.

### Inclusion and exclusion criteria

The inclusion criteria for this study included English or French-language, peer-reviewed, observational studies of adult (age 18+ years) residents of the 16 countries in the United Nations (UN) West African subregion: Benin, Burkina Faso, Cabo Verde, Cote d’ Ivoire, The Gambia, Ghana, Guinea, Guinea-Bissau, Liberia, Mali, Mauritania, Niger, Nigeria, Senegal, Sierra Leone and Togo. Eligible research designs included quantitative, qualitative or mixed methods. Publications deriving from the same study sample were included only if they provided additional results pertinent to this systematic review.

Exclusion criteria included studies published prior to 2007 (the release year of the IPCC’s Fourth Assessment Report), studies not written in English or French, studies outside of the UN West Africa subregion, studies of children age < 18 years, conference abstracts or proceedings, protocols, frameworks, commentaries, articles in media, editorials, literature reviews without a search strategy, letters to the editor, replies from author and erratum. Non-peer-reviewed studies were also excluded.

### Screening and selection

The senior author and a trained research assistant independently screened titles and abstracts of studies to identify relevant studies. Any discrepancies and disagreements between screeners were resolved through discussion.

### Data extraction and synthesis

Data extraction was completed independently by the senior author and two research assistants. The data were extracted for this review using a standardised and piloted data extraction form and were as follows: author, publication year, country, methodological approach, data collection methods, study period, sample size, age range of study participants, gender distribution of study participants, extreme weather event type and mental health outcome. Any discrepancies and disagreements between data extractors were resolved by discussion.

Data were synthesised through the creation of detailed tables, with included studies represented in rows and study data in columns. Given the heterogeneity of the methods and analyses of the selected studies, a meta-analysis was not performed for this analysis. As an alternative, we provided a qualitative narrative overview of the results describing the mental health impacts of extreme weather events in West Africa.

### Quality appraisal

The critical appraisal skills programme (CASP) was used to review qualitative studies.^27^ This tool includes 10 criteria questions regarding study design, sampling, data collection, reflexivity, ethical issues, data analysis, findings and value of the research. The studies were rated as high (meeting 9–10 criteria), medium (meeting 7–8 criteria) or low (meeting ≤ 6 criteria) quality.

We selected the CHANGE (Climate Health ANalysis Grading Evaluation) tool for the quantitative studies because it was developed specifically for weight-of-evidence reviews at the intersection of climate change and health, making it well suited for this review.^28^ The CHANGE tool evaluated 13 criteria questions covering domains that can produce biases in individual studies, such as lack of transparency, selection bias, bias in exposure, outcome and confounder measurement, and bias in the selection of reported results. Study domains were rated from 1 to 5, with a score of 1 indicating high scientific rigour, a score of 4 indicating poor scientific rigour and a score of 5 for domains that were not applicable. For mixed-methods studies, the qualitative and quantitative components were appraised separately using the CASP and CHANGE tools, respectively. Where a study’s two components diverged in quality, we weighed its contribution according to the rating of the component from which a given finding was drawn, rather than assigning the study a single overall quality designation.

The methodological quality assessment of all articles was independently conducted by two trained research assistants, and any discrepancies were discussed and resolved by consensus. Because this review used a systematic narrative synthesis rather than meta-analysis, findings were not statistically pooled. Instead, we interpreted findings in relation to study design, methodological quality and relevance to the review question. Studies rated more highly on the applicable appraisal tool were given greater weight in shaping the synthesis and conclusions. Where findings were based on studies with identified methodological limitations, we tempered the strength of claims accordingly. When multiple publications drew on the same underlying study sample but reported distinct outcomes or analytic questions, we treated them as separate reports of a single study sample rather than as independent studies.

## Review findings

### Search results

A total of 2968 peer-reviewed articles were identified through the database search, of which 13 met the criteria for inclusion in this review. Inter-reviewer agreement for study inclusion was strong (Cohen’s κ = 0.72). Figure 1 displays the PRISMA flowchart, detailing the criteria employed in the article selection process.

**FIGURE 1:**
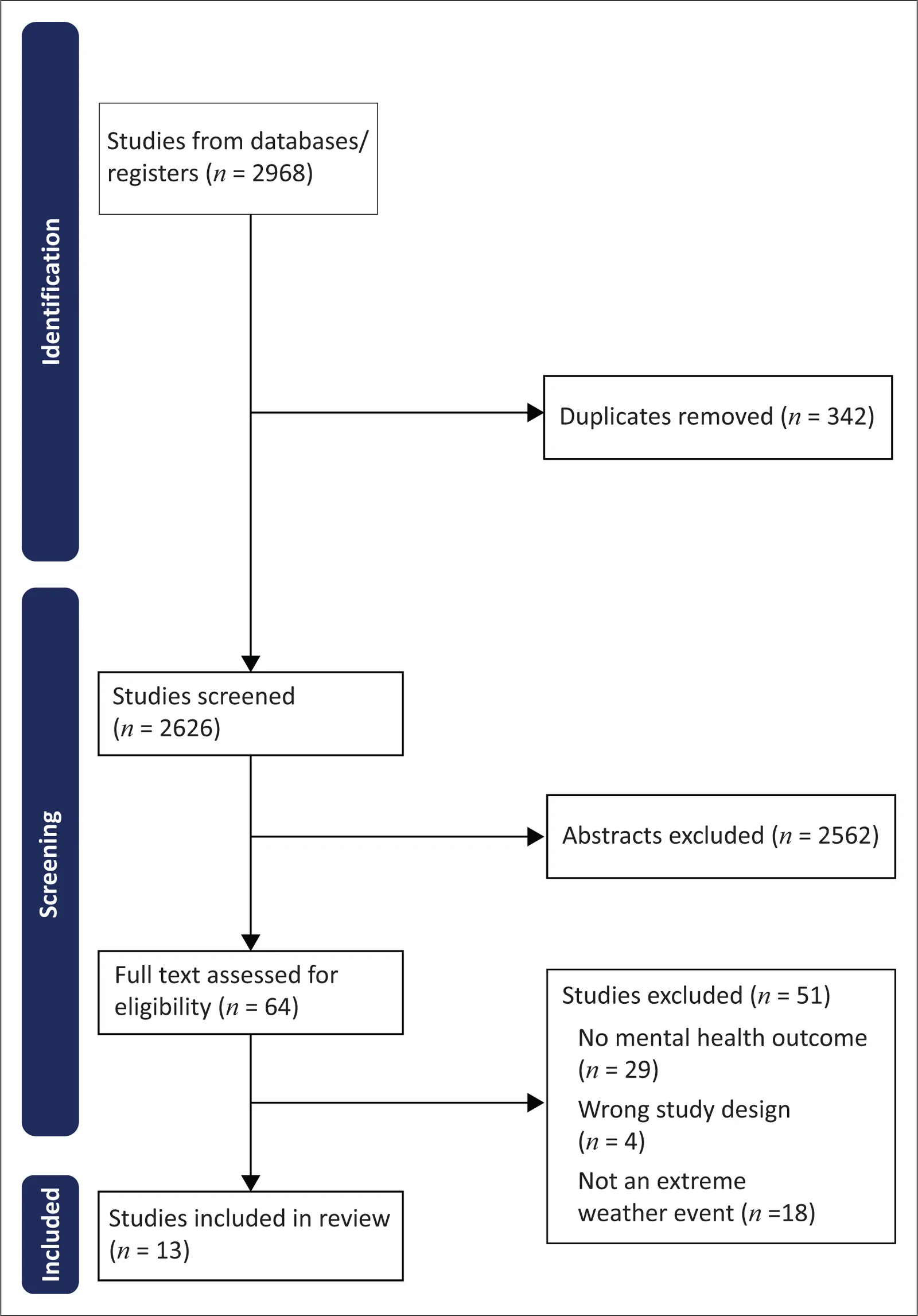
Preferred Reporting Items for Systematic Reviews and Meta-Analyses flow diagram.

### Study characteristics

Table 1 presents the characteristics of the 13 studies, including two publications from the same study sample that met inclusion criteria.^29,30^ Studies are presented in alphabetical order by the first author’s last name. Eight of these studies were based in Ghana,^29,30,31,32,33,34,35,36^ with the remaining based in Nigeria (*n* = 2),^37,38^ Sierra Leone (*n* = 1)^39^ and Burkina Faso (*n* = 2).^40,41^ Seven articles reported on qualitative research, four articles reported on quantitative research and two reported on mixed-methods research. The duration of data collection ranged from 1 month to 25 months, and one study did not include the time period of data collection.^34^ Data collection methods included questionnaires (*n* = 6),^29,30,32,34,37,38^ interviews (*n* = 5),^32,35,36,37,40,41^ focus groups (*n* = 4),^32,35,36,37,40,41^ photovoice (*n* = 1)^31^ and participatory mapping (*n* = 1).^33^

**TABLE 1:** Characteristics of the included studies (*N* = 13).

Author(s), year	Country	Setting	Methodological approach	Data collection method(s)	Study period	Sample size	Age range	Gender	Extreme weather event(s)	Mental health impact(s)	Key findings
Abass et al., 2022^29^^†^	Ghana	Urban: Kumasi; Rural: Central Gonja District	Quantitative	Questionnaire	July 2017– August 2017	767	20 – 91 years	39% female	Flooding	Psychological distress^‡^	Flood exposure was positively associated with psychological distress in the full sample (*p* < 0.005), with stronger associations among women (*p* < 0.001) and younger adults (*p* < 0.001).
Abass et al., 2023^30^^†^	Ghana	Urban: Kumasi; Rural: Central Gonja District	Quantitative	Questionnaire	July 2017 – August 2017	767	20 – 91 years	39% female	Flooding	Mental distress^‡^	Flood health-risk perception mediated 27% of the association between flood stressors and mental distress.
Adams and Nyantakyi-Frimpong, 2021^31^	Ghana	Urban: Old Fadama; Urban: Accra	Qualitative	Photovoice	June 2018 – June 2020	20	18 – 55 years	25% female	Flooding	Acute anxiety; chronic stress	Recurrent flooding was associated with adverse mental health outcomes Acute anxiety and chronic stress were most prominent themes.
Ajibade et al., 2013^37^	Nigeria	Urban: Ijora – Badia; Urban: Ajah; Urban: Victoria Island areas in Lagos, Lagos State	Mixed	In-depth interview Questionnaire Focus group	May 2011 – November 2011	453	≥ 18 years	100% female	Urban flooding	Anxiety; psychological trauma/stress	Personal health problems were reported by 57% of women in low-income neighbourhoods versus 7% among women in high-income neighbourhoods (*p* < 0.01).
Akoteyon, 2022^38^	Nigeria	Urban: Lagos	Quantitative	Questionnaire	October 2018 – November 2018	400	Not reported	38% female	Flooding	Emotional and psychological stress	Emotional and psychological stress was identified by respondents as a perceived flood risk. Emotional and psychological stress prevalence ranged from 48% to 82%.
Asamoah et al., 2024^35^	Ghana	Rural: White Volta Basin	Qualitative	In-depth interview Focus group	September 2020	45	≥ 18 years	42% female	Flooding	PTSD symptoms; anxiety; chronic stress; depression	Participants reported long-term PTSD-like symptoms, anxiety, chronic stress and depression related to recurrent flooding.
Bangura et al., 2013^39^	Sierra Leone	Rural: Kambia District, North West Province; Rural: Kono District, Eastern Province	Qualitative	Facilitated group discussion	March 2011 – September 2011	250	Not reported	Not reported	Increased temperatures Severe storms Wild bush fires Flood events	Trauma	Participants reported that severe storms were associated with being ‘traumatised’ with fear over food shortages and loss of homes and possessions.
Boafo and Yeboah, 2024^36^	Ghana	Rural: Chepuri	Qualitative	In-depth interview Focus group	August 2023 – September 2023	36	50 – 77 years	Not reported	Drought Flooding	Anxiety; sadness; depression/ despair	Loss of livelihoods produced ecological grief manifested as sadness, anxiety and despair.
Kadio et al., 2024^41^	Burkina Faso	Urban: Bogodogu; Rural: Kaya	Qualitative	In-depth interview Focus group	October 2020	71	20 – 40 years	91% female	Increased temperatures	Mental well-being; sadness; stress; anxiety	Extreme heat affected pregnant and postpartum women’s mental well-being, with participants reporting sadness and anxiety.
Nuvey et al., 2020^32^	Ghana	Rural: Bunkpurugu- Yunyoo District, Northern Region; Rural: Kwahu Afram Plains South District, Eastern Region	Mixed	Questionnaire Guided interview	July 2018 – September 2018	287	19 – 78 years	7% female	Drought	Depression; anxiety; Stress^‡^	Sixty per cent of livestock farmers had poor mental health (72% depressed, 66% anxious, 59% stressed); mental well-being was negatively associated with number of adverse events, proportion of livestock lost, emotional attachment to livestock and self-reported physical illness, but positively associated with herd size (all *p* < 0.05)
Osman et al., 2023^34^	Ghana	Urban: Old Fadama	Quantitative	Questionnaire	Post 2021 flood event, date not reported	386	≥ 18 years	Not reported	Flooding	Depression^‡^	Depression severity linked to damaged household items, with damaged furniture/chairs/tables/sofa producing more severe depression (e.g. vs Electronic Fan/Iron, *p* < 0.01).
Sorgho et al., 2020^40^	Burkina Faso	Rural: Nouna, Kossi Province	Qualitative	In-depth interview	October 2018 – November 2018	32	28 – 80 years	0% female	Drought Flooding	Stress; mental health difficulties	Subsistence farmers expressed constant stress, worry, anxiety and fear centred on household food security, with inability to adapt to climate change associated with mental health difficulties.
Tschakert et al., 2013^33^	Ghana	Urban: Greater Accra Region; Rural: Northern Region; Rural: Upper West Region	Qualitative	Participatory mapping	June 2008 – July 2010	112	20 – 85 years	23% female	Drought	Solastalgia; sadness	Drying up of water sources, failing agriculture and deforestation was associated with acute sadness and helplessness among those left behind in ‘hollow homes’, interpreted as solastalgia; extreme sadness was the most frequently elicited emotion.

Note: Please see the full reference list of the article Nwanaji-Enwerem JC, Perez-Cruet JM, Sprague N, et al. Extreme weather events and mental health impacts in West Africa: A systematic review of evidence gaps. J Public Health Africa. 2026;17(1), a1760. https://doi.org/10.4102/jphia.v17i1.1760, for more information

PTSD, post-traumatic stress disorder.

† , Publications based on same study sample;

‡ , Study employed a validated psychometric instrument: the Kessler psychological distress scale (K10) in Abass et al. (2022 and 2023)^29,30^; the Depression Anxiety and Stress Scale (DASS-21) in Nuvey et al. (2020)^32^; or the Patient Health Questionnaire (PHQ-9) in Osman et al. (2023).^34^

The studies included participants from both urban (*n* = 4) and rural (*n* = 5) settings, with four studies sampling from both settings.^29,33,41^ Sample sizes ranged from 20 to 767 study participants. Across the studies, most study participants were male, ranging from 51% to 100% male. One study was exclusively female,^37^ and three studies did not specify a gender distribution.^34,36,39^ Ten studies focused exclusively on the impacts of one extreme weather event,^29,30,31,32,33,34,35,37,38,41^ including seven studies of flooding, two studies of drought and one study of extreme heat. Three studies focused on multiple extreme weather events: a Sierra Leone-based study focused on increased temperatures, severe storms, wild bushfires and flood events,^39^ and studies based in Ghana and Burkina Faso focused on drought and flooding.^36,40^

### Mental health impacts

#### Flooding impacts

Abass et al.^29^ examined the psychological impact of flood exposure on adults residing in flood-prone urban and rural areas of Ghana. The study employed an interviewer-administered questionnaire that included a psychological distress index to quantify the mental health challenges faced by individuals in the aftermath of floods, such as anxiety, depression and other stress-related symptoms. Study results indicated a significant association between flood exposure and psychological distress among participants, with women showing a higher degree of distress compared to their male counterparts. Furthermore, younger adults reported stronger associations between flood exposure and psychological distress than older adults. Their 2023 article with the same sample reported a strong association between flood stressors and mental distress, assessed using the Kessler psychological distress scale.^30^ Findings also suggested that the relationship between flood stressors and risk of mental distress was partially explained by individual flood health-risk perceptions.

Akoteyon^38^ explored the impacts of flooding in Lagos, Nigeria, by administering questionnaires to 400 residents across four settlements with the objective of gathering data on resident experiences and perspectives regarding flooding and its management. Using a single Likert-scaled question, the study identified emotional and psychological stress as one of the significant flood impacts experienced by residents in the study area, with observed prevalences ranging from 48% to 82% across different settlements. In addition to mental stress among the population, flooding impacts included poor sanitation, increased mosquito breeding sites and water contamination leading to waterborne diseases, highlighting the multifaceted challenges of flooding in urban settings.

Adams and Nyantakyi-Frimpong^31^ explored how repeated instances of flooding resulted in uneven impacts on mental health in Ghana. Women and children experienced greater vulnerability to mental health impacts. These impacts included acute anxiety, chronic stress and a threat to sense of place. Social characteristics such as poor housing conditions, low socioeconomic status, food insecurity, large family size and poor social cohesion contributed significantly to shaping increased vulnerability for these subgroups.

Ajibade et al.^37^ delved into the impact of flooding on urban women in different income areas of Lagos, Nigeria. The study found that women in low-income areas faced more severe consequences such as increased illnesses, displacement and loss of assets compared to those in middle- and high-income areas. Women in low-income areas experienced heightened anxiety and stress, and a diminished sense of peace during floods, primarily due to perceived threats to their children’s health and housing conditions. Those who evacuated faced additional concerns regarding privacy. Factors hindering recovery included lack of social assistance, limited financial resources, weak social networks and the inability to relocate.

Asamoah et al.^35^ examined the impacts of flooding on communities in Northern Ghana. Immediate effects included crop destruction, damage to homes, increased health risks and disruptions to education. Flooding also intensified economic hardships, prompting out-migration to cities in search of work and early child marriages as a means of securing dowries for financial relief. The health impacts of dam-related flooding were assessed through focus group discussions and key informant interviews conducted in flood-prone communities. Long-term mental health consequences, such as anxiety, depression and post-traumatic stress disorder, were commonly reported by affected individuals.

Osman et al.^34^ sampled 386 heads of households within the Old Fadama, Accra, Ghana, community to assess depression levels associated with flood-damaged household items. The study assessed depression levels among participants using the Patient Health Questionnaire (PHQ-9). Study findings indicated significant differences in depression levels based on the type of household item damaged, with furniture (chairs, tables and sofas) being ranked highest in terms of causing depression. Items with moderate costs generally induced mild levels of depression, whereas more affordable items were less likely to result in severe depression. The results of this study indicated the need for flood insurance policies, identification of valued and hard-to-replace items and tailored psychological support for flood victims particularly in informal settlements to mitigate depression.

#### Drought impacts

Nuvey et al.^32^ examined the mental health impacts of drought among farmers in Ghana. The Depression, Anxiety and Stress Scale – 21 (DASS-21) was used to assess mental health. Approximately 60% of livestock farmers had poor mental health according to the DASS-21 scale, and most farmers classified themselves as being depressed, anxious or stressed. The authors reported a negative association of mental well-being with the number of adverse events experienced, proportion of livestock lost, emotional attachment to livestock and self-reported physical illness.

Tschakert et al.^33^ explored the heat and drought experiences of individuals from urban Accra, Ghana, as well as those rural farming areas in Northern and Upper West Regions of Ghana. Findings highlighted the emotional impact of ongoing environmental changes on individuals’ well-being. The term ‘solastalgia’ was used to describe the homesickness and distress felt by people witnessing the persistent environmental deterioration in their home areas. The interconnected effects of climate- and migration-induced changes over several decades also led to a loss of social connections, contributing to the overall emotional toll on individuals. The study emphasises that these emotional responses, including extreme sadness, grief and uneasiness, are more strongly connected to long-term climate-related changes, like drought, rather than immediate events like floods or fires.

#### Extreme heat impacts

Kadio et al.^41^ examined the multifaceted impacts of extreme heat on pregnant and postpartum women in Burkina Faso. Using in-depth interviews, focus group discussions and key informant interviews, the study found that extreme heat significantly affected women’s well-being and daily functioning. Postpartum mothers reported increased anxiety due to their babies’ discomfort from the heat. Socially, heat strained interpersonal relationships, leading to isolation, family discord and reduced intimacy with spouses. Cultural practices that required mothers and newborns to remain indoors during the postpartum period intensified these challenges. Economically, women’s productivity declined as they avoided physically demanding tasks during periods of extreme heat, leading to reduced household income. The findings also revealed gaps in awareness of heat-related health risks among both women and healthcare professionals in Burkina Faso.

#### Impacts of multiple extreme weather events

Sorgho et al.^40^ focused on farmers’ perceptions of flooding and drought events and its impact on health. Study findings highlighted the constant concern among household heads regarding self-sufficiency and the threat of hunger, leading to ongoing stress and anxiety that affected their mental well-being. Participants identified family and friends within and outside their community, along with support from non-governmental organisations (NGOs) and provincial government services, as crucial sources of assistance.

Bangura et al.^39^ reported on the impacts of weather changes in rural Sierra Leone. The unpredictability of the rainy season, declining crop yields, food scarcity and income insecurity contributed to heightened stress, anxiety and uncertainty among study participants. The inability to predict weather events and the resulting financial strain on farming households led to feelings of helplessness and frustration. The trauma experienced by individuals affected by severe storms had strong psychological impacts, such as fear of food shortages and loss of essential possessions. Additionally, the study found that extended periods of scarcity intensified emotional distress in communities, contributing to violence, disrespectful behaviour and power struggles within households.

Boafo and Yeboah^36^ explored ecological grief as a response to drought- and flood-related ecological loss among smallholder farmers in Ghana’s Upper West Region. In-depth interviews and a focus group discussion revealed that farmers experienced feelings of helplessness, hopelessness, anxiety, sadness and despair due to extreme weather events, which disrupted agricultural activities and led to crop failures. The study identified three primary sources of ecological grief: the loss of livelihoods, the loss of indigenous seeds and genetic resources and the loss of indigenous ecological knowledge. The findings highlight that ecological grief manifests not only as individual emotional distress, but also as collective mourning within communities facing similar losses.

### Quality appraisal

Of the nine studies that employed qualitative methods, five were rated as high quality,^35,37,39,40,41^ three were rated as medium quality,^31,33,36^ and one was rated as low quality^32^ (Table 2). None of the qualitative studies reported on the relationship between the researchers and study participants.

**TABLE 2:** Critical appraisal of qualitative studies (*n* = 9†).

Quality criteria	Are the results of the study valid?						What are the results?			Will the results help locally? – How valuable is the research?	Quality rating (CASP score)

	Was there a clear statement of the aims of the research?	Is a qualitative methodology appropriate?	Was the research design appropriate to address the aims of the research?	Was the recruitment strategy appropriate to the aims of the research?	Was the data collected in a way that addressed the research issue?	Has the relationship between the researcher and participants been adequately considered?	Have ethical issues been taken into consideration?	Was the data analysis sufficiently rigorous?	Is there a clear statement of findings?		
Adams and Nyantakyi-Frimpong, 2021^31^	Yes	Yes	Yes	No	Yes	No	Yes	Yes	Yes	Yes. Engages a community that is often neglected by authorities.	Medium (8)
Ajibade et al., 2013^37^	Yes	Yes	Yes	Yes	Yes	No	Yes	Yes	Yes	Yes. Discusses implications for a disaster response that considers the intersectional identities of women.	High (9)
Asamoah et al., 2024^35^	Yes	Yes	Yes	Yes	Yes	No	Yes	Yes	Yes	Yes. Engaged disaster stakeholders in understudied region.	High (9)
Bangura et al., 2013^39^	Yes	Yes	Yes	Yes	Yes	No	No	Yes.	Yes	Yes. Focuses on differences in women’s perspectives.	High (9)
Boafo and Yeboah, 2024^36^	Yes	Yes	Yes	Yes	Yes	No	No	Yes	Yes	Yes. Discusses manifestations of ecological grief among farmers.	Medium (8)
Kadio et al., 2024^41^	Yes	Yes	Yes	Yes	Yes	No	Yes	Yes	Yes	Yes. Focuses on maternal well-being.	High (9)
Nuvey et al., 2020^32^	No	Yes	No	Yes	Yes	No	Yes	No	Yes	Yes. Understudied region.	Low (6)
Sorgho et al., 2020^40^	Yes	Yes	Yes	Yes	Yes	No	Yes	Yes	Yes	Yes. Robust discussion of policy implications.	High (9)
Tschakert et al., 2013^33^	Yes	Yes	Yes	Yes	Yes	No	No	Yes	Yes	Yes. Participatory mapping provided additional context.	Medium (8)

Note: Please see the full reference list of the article Nwanaji-Enwerem JC, Perez-Cruet JM, Sprague N, et al. Extreme weather events and mental health impacts in West Africa: A systematic review of evidence gaps. J Public Health Africa. 2026;17(1), a1760. https://doi.org/10.4102/jphia.v17i1.1760, for more information

CASP, critical appraisal skills programme.

† , Includes two mixed-methods studies.

The average score of the six studies that employed quantitative methods was 2.1, which is considered moderate quality (Table 3).^28^ The study domains of selection bias (mean score = 1.2) and selective reporting bias (mean score = 1) were scored as high quality. The domains of transparency (mean score = 3.0) and detection bias (mean score = 3.3) were scored as poor quality. Most of the quantitative studies failed to provide detailed exposure characterisations of the extreme weather event, particularly the frequency and duration of the event. Notably, the more recent studies had larger sample sizes and higher quality scores than studies published before 2022.

**TABLE 3:** Critical appraisal of quantitative studies (*n* = 6†).

Quality criteria	Abass et al., 2022^29^	Abass et al., 2023^30^	Ajibade et al., 2013^37^	Akoteyon, 2023^38^	Nuvey et al., 2020^32^	Osman et al., 2023^34^
**Transparency (mean score)**	**3**	**3**	**3**	**2.5**	**3.3**	**3**
1. Does the study clearly specify the research question?	2	2	2	2	3	2
2. Does the study clearly state the inclusion and exclusion criteria?	4	4	4	4	4	4
3. Is the research reproducible?	2	2	2	2	2	2
4. Does the study clearly assess the ‘risk of bias?’	4	4	4	2	4	4
**Selection bias (mean score)**	**1**	**1**	**1**	**1**	**2**	**1**
5. Are the individuals selected to participate in the study likely to be representative of the target population?	1	1	1	1	2	1
**Covariate variable selection (mean score)**	**1**	**1**	**3**	**2**	**4**	**1**
6. Did the study design or analysis account for the minimally sufficient set of confounding and covariate variables	1	1	3	2	4	1
**Detection bias (mean score)**	**3**	**2.7**	**3.3**	**3**	**3.8**	**3.8**
7. Was the measurement ascertainment of the climate variable well described with detail of the technology, developer, detailed usage and measurement error of the sensor?	4	4	4	4	4	4
8. Was the measurement ascertainment of the climate variable well described with detail of the frequency and recording of location updates and the method, period and duration of data collection clearly specified?	3	3	3	3	4	4
9. Was the climate variable well described with detail of the data availability?	3	2	4	2	4	4
10. Was there a clear justification for the chosen climate exposure and the method of exposure assessment?	1	1	1	1	4	3
11. Can we be confident in the exposure characterisation?	4	4	4	4	4	4
12. Were confounding variables assessed consistently across groups using valid and reliable measures?	3	2	4	4	3	5
**Selective reporting bias (mean score)**	**1**	**1**	**5**	**1**	**1**	**1**
13. Were all measured outcomes (based on the research aims) reported?	1	1	5	1	1	1
**Overall quality rating (mean score of subcategories) ‡**	**1.8**	**1.7**	**2.4**	**1.9**	**2.5**	**2.0**

Note: Please see the full reference list of the article Nwanaji-Enwerem JC, Perez-Cruet JM, Sprague N, et al. Extreme weather events and mental health impacts in West Africa: A systematic review of evidence gaps. J Public Health Africa. 2026;17(1), a1760. https://doi.org/10.4102/jphia.v17i1.1760, for more information

† , Includes two mixed-methods studies;

‡ , Scores range from 1 to 5, with lower scores indicating higher quality. Domains rated 5 (Not Applicable) were excluded from mean scores.

## Discussion

This systematic review identifies three key themes: Firstly, while the direct effects of extreme weather events on mental health are often emphasised, these events may also indirectly impact mental health through pathways related to food insecurity, physical health deterioration and zoonotic disease exposure. Secondly, certain populations, such as women and low-income groups, appear to experience differential impacts. Thirdly, policy opportunities, such as expanding mental health services and strengthening social safety nets, may be useful for mitigating the mental health burden of climate-related weather events and supporting the most affected populations.

One major theme of the reviewed articles was that extreme weather events may have both direct and indirect effects on mental health. Indeed, floods or droughts themselves could be a source of distress in the included studies, but the indirect effects of extreme weather events provided additional cultural insights. For instance, Abass et al.^30^ performed a mediation analysis and demonstrated that flood health-risk perception mediated 27% of the association of flood stressors with mental distress in Ghanian community-dwelling adults. Nuvey et al.^32^ provided the example of livestock impacts as a way that these events could have indirect effects on the mental health of farmers in Ghana. The authors repeatedly highlighted how climate disasters like droughts or floods could contribute to crop destruction and livestock disease and death, resulting in food insecurity that could worsen farmers’ mental well-being. Losses from the death of livestock could then result in the stressor of additional food insecurity and even worse mental well-being in farmers. Even if farmers sold diseased cattle at meat markets to recoup losses, consumption of diseased meat could contribute to poor physical health in the population, which is also associated with worse mental well-being. The results from Bangura et al.^39^ further suggested the connection between climate, physical health and mental well-being. In their study of individuals living in rural Sierra Leone, climate-related livelihood insecurity was associated with prostitution, crime, violence and child labour. Each of these scenarios can contribute to stressful experiences like divorce, household breakdown and carry physical health risks that can simultaneously impact mental well-being. Sorgho et al.^40^ mentioned how decreased income due to extreme weather events could limits one’s capacity to address health needs. Similarly, the work by Ajibade et al.^37^ highlighted the disruption of coping mechanisms (e.g. social networks, financial resources, etc.) as another indirect way that climate could adversely impact mental well-being.

Importantly, the articles also demonstrated that beyond direct and indirect effects, truly understanding the effects of climate change on mental health required an appreciation of different vulnerabilities. The most robust representation of these different vulnerabilities was in the articles that highlighted differential climate effects between men and women study participants and those of differing socioeconomic status. In a sex-based analysis, Abass et al. reported that the magnitude of the association between flood exposure and psychological distress was greater for women than men. The authors believed that the disproportionate impact on women was related to their social position, including social suppression and lower levels of education.^29^ Kadio et al.^41^ observed that pregnant and postpartum women faced heightened mental health vulnerabilities under extreme heat, exacerbated by cultural practices (e.g. postpartum confinement indoors) and economic pressures from reduced work productivity. These intersecting stressors, rooted in gender roles, limited healthcare awareness and livelihood disruptions, underscore women’s potential disproportionate psychological burdens in climate-vulnerable settings. Bangura et al.^39^ further commented that some of the disproportionate impacts experienced by women could be due to gender roles as women often bear the burden of caring for children, but also the elderly and the sick even during times of disaster. Ajibade et al.’s work expands upon this assertion by reporting on how socioeconomic status could further impact the vulnerability of women. Specifically, they report that women from low-income Lagos neighbourhoods demonstrated greater flood-related adverse impacts and slower recovery when compared to women from higher-income neighbourhoods.^37^ Osman et al.^34^ identified socioeconomic status independent from sex as another important vulnerability as persons of lower education in their cohort were more likely to experience severe levels of flood-related depression. Apart from sex and socioeconomic status differences, Abass et al.^29^ report different vulnerabilities with age, as the flood and distress relationship was more robust among younger individuals (20–59 years of age) when compared to their older counterparts (ages 60 years and above). There was no clear reason for this observed age difference, and the authors note that most of the existing literature supports older individuals being more impacted.

The reviewed articles also noted limits to individual resilience or ability to cope with the effects of climate change and alluded to a series of broader policy interventions that may prove to be beneficial. A major takeaway from the aforementioned discussion on sex vulnerabilities is the potential for sex-targeted policies or philanthropic initiatives to support women and other disproportionately impacted groups during disasters.^37^ Akoteyon^38^ emphasised that several of the sites of flooding and property losses in Lagos State, Nigeria, were areas of land that were developed without a proper environmental impact assessment (EIA). Thus, conducting and properly enforcing EIAs represents one potentially actionable target for future collective efforts. Adams et al. and Abass et al. echo the concern for rapid, uncontrolled development as a major contributing factor. These authors offer greater emphasis in municipal planning (e.g. increased greenspace allocation, appropriate drainage systems, sufficient waste management systems, etc.) as a potentially fruitful local policy focus.^30,31^ Work by Osman and colleagues demonstrated that the relationship between flood exposure and depression could be modified by what personal property was damaged by a flood. They found that the level and cost of damaged items influenced the levels of depression participants reported.^34^ These findings suggest that policies or insurance schemes aimed at replacing or supporting people who may have lost these impactful items is a possible intervention. Osman et al. also called for Ghana’s National Disaster Management Organisation (NADMO) to include psychological assistance provisions for individuals impacted by floods. Nuvey et al.^32^ called for more animal and human vaccination campaigns to help safeguard against climate-related zoonoses. Finally, Sorgho et al.^40^ highlighted the lack of formal education on climate topics among other areas of education as an issue requiring attention. Consequently, efforts to enhance individual and population understanding of why extreme weather events occur and their specific risks may prove beneficial in the West African region. Not explicitly mentioned in the articles, but likely to be important to addressing the impacts of climate on mental health, are investments in research infrastructure (e.g. funding, data collection capacity) and bolstering mental health clinical capacity given that there is a shortage of mental health professionals in the region.

Our review revealed several gaps in the literature, particularly a paucity of research on adult mental health and extreme weather events in West Africa. Although our review provides important insights on mental health impacts, it does have some limitations. Firstly, the included studies are from only four of 16 West African countries, which poses some limitations to the generalisability of the findings. Secondly, in contrast to symptoms of general distress, clinically diagnosed disaster-related mental health disorders (e.g. PTSD) were rarely investigated in the included studies. The studies included in our review represent a mix of quantitative and qualitative research approaches, and the majority are of high and moderate quality, but there remains a need for research with increased country representation, and a full range of mental health impacts, to truly capture outcomes that are representative of the entire region. Additionally, for mixed-methods studies, the qualitative and quantitative components sometimes differed in methodological quality; in these cases, we interpreted each component’s findings in light of its respective appraisal rather than treating the study as uniformly strong or weak. Thirdly, according to the United Nations International Children’s Emergency Fund (UNICEF),^42^ almost half of Africa’s present population is under the age of 18 years. Our review focuses on individuals greater than 18 years of age. As such, our study findings may not be generalisable to this substantial proportion of the West African population. Given that mental health impacts of climate exposures may differ between adults and children, who are in their earlier stages of development, there is further impetus for future research that is centred on the mental health experiences of children in the context of extreme weather events in West Africa. Fourthly, most of the studies focused on flooding, and there remains a need for studies that examine other weather-related exposures. Fifthly, many of the included studies are cross-sectional in nature and cannot speak to causal relationships. Studies with longitudinal data are needed to better elucidate temporal relationships. Sixthly, heterogeneity in mental health outcome assessment, including the use of different validated instruments and variation in diagnostic frameworks across settings, may limit direct comparability of findings across studies. Seventhly, although we intentionally focused on the West African region, some may view this geographic emphasis as limiting the broader generalisability of our findings. However, we argue that this regional focus has allowed us to propose more context-specific solutions to address the mental health impacts of climate change, while still identifying areas of overlap with challenges and approaches observed in other regions.

For example, studies from Australia have described feelings of hopelessness among farmers facing drought conditions,^43^ mirroring similar sentiments reported by farmers in Ghana confronting climate-related stressors.^32^ Likewise, a review from Latin America and the Caribbean highlighted how climate-related mental distress can indirectly contribute to rising rates of noncommunicable diseases such as obesity and diabetes, conditions that, in turn, exacerbate psychological distress.^44^ This was among the indirect effects that we observed from our analysis. That same Latin America and Caribbean-based review emphasised the mental health toll of climate-related migration, particularly due to the loss of community and social ties, an issue also identified in two of our included studies from Ghana.^33,35^ These parallels suggest that, despite geographic differences, shared lessons and strategies may be valuable across regions. Additionally, a review on extreme weather and mental health in South and Southeast Asia identified women and individuals of lower socioeconomic status as particularly vulnerable populations,^45^ consistent with the themes in our West African review. The same study also pointed to pre-existing psychological conditions and past trauma as risk factors, elements not explicitly surfaced in our findings, but which warrant further investigation in future West African research. Notably, the Asia-based review emphasised the importance of longitudinal data to better characterise causal relationships. Given that most studies we identified were cross-sectional, we echo the call for longitudinal research to strengthen the evidence base in West Africa. Finally, this review protocol was not registered in PROSPERO (International Prospective Register of Systematic Reviews) or another registry, which we acknowledge as a limitation.

## Conclusion

In conclusion, the range of cultural and geographic differences across the African continent prompted our focus on the region of West Africa to allow for a more nuanced understanding of any observed relationships. We believed that a nuanced understanding would also improve the chances of actionable solutions-oriented idea generation. In this systematic review examining relationships of extreme weather events and adult mental health in West Africa, we screened over 2900 articles and identified 13 that met inclusion criteria. These articles suggested direct and indirect relationships of climate events with mental health and highlighted populations that may be particularly vulnerable to these effects including women and persons of lower socioeconomic status. The articles also provide insights to potentially address these climate and health challenges. Possible solutions include policies to support women, targeted psychological assistance provisions, vaccination campaigns to reduce climate-related zoonoses, reforms to building and development mechanisms, more robust property insurance programmes and broader population climate health education. Continued investments in research infrastructure and mental health clinical capacity may also be important for addressing these dire needs.
